# Exploratory analysis of protein translation regulatory networks using hierarchical random graphs

**DOI:** 10.1186/1471-2105-11-S3-S2

**Published:** 2010-04-29

**Authors:** Daniel D Wu, Xiaohua Hu, EK Park, Xiaofeng Wang, Jiali Feng, Xindong Wu

**Affiliations:** 1 College of Information Science and Technology, Drexel University, Philadelphia, Pennsylvania, USA; 2 CSI-CUNY, Staten Island, NY 10314, USA; 3 College of Information Engineering, Shanghai Maritime University, Shanghai, China; 4 Department of Computer Science, University of Vermont, Vermont, USA; 5 School of Computer Science and Information Engineering, Hefei University of Technology, Hefei 230009, China

## Abstract

**Abstract:**

**Background**

Protein translation is a vital cellular process for any living organism. The availability of interaction databases provides an opportunity for researchers to exploit the immense amount of data *in silico* such as studying biological networks. There has been an extensive effort using computational methods in deciphering the transcriptional regulatory networks. However, research on translation regulatory networks has caught little attention in the bioinformatics and computational biology community.

**Results**

In this paper, we present an exploratory analysis of yeast protein translation regulatory networks using hierarchical random graphs. We derive a protein translation regulatory network from a protein-protein interaction dataset. Using a hierarchical random graph model, we show that the network exhibits well organized hierarchical structure. In addition, we apply this technique to predict missing links in the network.

**Conclusions**

The hierarchical random graph mode can be a potentially useful technique for inferring hierarchical structure from network data and predicting missing links in partly known networks. The results from the reconstructed protein translation regulatory networks have potential implications for better understanding mechanisms of translational control from a system’s perspective.

## Background 

The central dogma of molecular biology describes that the genetic information is transferred from DNA to mRNA through transcription and from mRNA to protein via translation. In every living organism, translation is a vital cellular process in which the information contained in the mRNA sequence is translated into the corresponding protein by the complex translation machinery. 

There are three major steps in protein biosynthesis: initiation, elongation, and termination. Initiation is a series of biochemical reactions leading to the binding of ribosome on the mRNA and the formation of the initiation complex around the start codon. This process involves various regulatory proteins (the so-called initiation factors). Eukaryotic protein synthesis exploits various mechanisms to initiate translation, including cap-dependent initiation, re-initiation, and internal initiation. For the majority of mRNAs in the cell, their translation is via the cap-dependent pathway. Although debatable, it is widely believed that some cellular mRNAs contain internal ribosome entry sites (IRES) and there exists a cap-independent, IRES mediated translation [1]. During elongation, codon-specific tRNAs are recruited by the ribosome to grow the polypeptide chain one amino acid at a time while the ribosome moves along the mRNA template (one codon at a time). This process also involves various elongation factors and proceeds in a cyclic manner. In termination, the termination codon is recognized by the ribosome. The newly synthesized peptide chain and eventually the ribosomes themselves are released [2].

Recent years have witnessed the breakthrough in high-throughput technologies that have been used in monitoring the various components of the transcription and translation machineries. DNA microarrays enable the estimation of the copy number for every mRNA species within a single cell and the changes in gene expression temporally or under different physiological conditions [3]. Two-dimensional gel electrophoresis coupled with tandem mass spectrometry makes it possible to measure simultaneously specific protein levels for thousands of proteins in the cell. These high-throughput technologies and the success of several genome projects are rapidly generating an enormous amount of data about genes and proteins that govern such cellular processes as transcription and translation. Analyzing these data is providing new insights into the regulatory mechanisms in many cellular systems. One of the major goals in post-genomic era is to elucidate in a holistic manner the mechanisms by which sub-cellular processes at the molecular level are manifest at the phenotypic level under physiological and pathological conditions.

The complexity and the large sizes of the transcription and translation machineries make computational approaches attractive and necessary in facilitating our understanding the design principles and functional properties of these cellular systems. Transcriptional regulation, used by cells to control gene expression, has been a focus in a variety of computational methods to infer the structure of genetic regulatory networks or to study their high level properties [4]. However, research on translational regulatory networks has caught little attention in the bioinformatics and computational biology community, either being underestimated or neglected. This contrast may partly due to two factors. Firstly, transcriptional control, other than translational control, has long been regarded by conventional wisdom as the primary control point in gene expression. Secondly, the success of genome projects and the application of high-throughput technologies provide tremendous amount of data about transcriptional regulation that are readily available for computational analysis. On the contrary, data about translational control are still probably too specialized so that they are consumed primarily by biologists.

Proteins, rather than DNAs or mRNAs, are the executors of the genetic program. They provide the structural framework of a cell and perform a variety of cellular functions such as serving as enzymes, hormones, growth factors, receptors, and signalling intermediates. Biological and phenotypic complexity eventually derives from changes in protein concentration and localization, post-translational modifications, and protein-protein interactions. Expression levels of a protein depend not only on transcription rates but also on such control mechanisms as nuclear export and mRNA localization, transcript stability, translational regulation, and protein degradation. Results from biological research have demonstrated that translational regulation is one of the major mechanisms regulating gene expression in cell growth, apoptosis, and tumorigenesis [5]. Therefore, study of protein translation networks, especially from computational systems biology approaches, may provide new insights into our understanding of this important cellular process.

Mehra and colleagues [6] develop a genome-wide model for the translation machinery in E. coli that provides mapping between changes in mRNA levels and changes in protein levels in response to environmental or genetic perturbations. They also propose a mathematical and computational framework [7] that can be applied to the analysis of the sensitivity of a translation network to perturbation in the rate constants and in the mRNA levels in the system. 

However, toward the goal of understanding how translation machinery functions from a system’s perspective that may enable us to form new theories and make new predictions, it is imperative that we have a better understanding of the structure and properties of protein translation networks. In pursuing such a goal, we previously reported a global analysis of network analysis of Protein Translation Regulatory Networks (PTRN) in yeast [8]. In this paper, we extend our efforts to study one important network feature: hierarchy. 

Biological processes are hierarchically organized, evident from interactions between molecules within a cell to relationships among members of an ecological system, and hierarchical structure plays an important role in the dynamics of these processes.

Active research has been done to assess whether a network is actually organized in a hierarchical manner and to identify the different levels in the hierarchy. The majority of the work has been focusing on identifying “global signatures” of a hierarchical network architecture. For example, Ravasz and colleagues [9] studied the hierarchical structure of metabolic networks and reported that the uncovered hierarchical modularity closely overlaps with known metabolic functions in E. coli. 

Out of many methods proposed to investigate the hierarchical organization in a network [10-14], an especially appealing one is the hierarchical random graph model introduced by Clauset and colleagues [13,14]. 

In the following, we define a PTRN that contains proteins involved in translational regulation and controls. We then describe the hierarchical random graph model and the adapted approach we use based on this model to infer the hierarchical structure of the constructed network and further to predict missing links within the network.

## Methods

### Datasets

The yeast protein-protein interactions data were downloaded from the General Repository for Interaction Datasets (GRID) [15]. We select GRID because it contains arguably the most comprehensive data. The GRID database includes all published large-scale interaction datasets as well as available curated interactions such as those deposited in BIND [16] and MIPS [17]. The yeast dataset we downloaded has 4,948 distinct proteins and 18,817 unique interactions. From this network, we derive the protein translation networks which contain proteins with MIPS functional categories related to protein translation as described next. 

### Construction of PTRN

We extract proteins that are involved in protein biosynthesis from MIPS functional category database as shown in Table 1. The extracted proteins belong to the following categories: 12.04 (translation), 12.04.01 (translation initiation), 12.04.02 (translation elongation), 12.04.03 (translation termination), and 12.07 (translational control). There are totally 133 unique proteins in this dataset. We then build the network by using protein-protein interaction data, including interactions among the selected proteins only and ignoring all other interactions. With the exclusion of the isolated proteins – those without any edges connecting to them – and self-looping interactions, the resulted network contains 108 vertices and 342 edges. 

**Table 1:** MIPS functional categories related to protein translation

Category	Description	# of Proteins
12.04	translation	88
12.04.01	translation initiation	40
12.04.02	translation elongation	21
12.04.03	translation termination	9
12.07	translational control	55

A protein may belong to more than one category. The number of proteins is the number of entries stored in each category.

There are several reasons for such a construction. First of all, our interest in this research has been focused on protein translation regulatory networks. Secondly, protein-protein interaction data are notorious noisy and incomplete. The approach we take allows us not only to study the hierarchy but also to predict missing links even with the noise and incompleteness in the background. At current stage, it is also more feasible computationally with networks of smaller sizes. In addition, we want to examine if hierarchical structure exists even in such isolated subnetworks. 

### Hierarchical random graphs

Our approach is based on a hierarchical random graph proposed by Clauset and colleagues [13,14], incorporating with work by Sales-Pardo and colleagues [12]. There are two important assumptions in this approach. Firstly, if a network has sub-networks with an equal probability connecting them, then the network can be represented by splitting off the sub-network off one at a time until the last one. Secondly, there may be more than one hierarchical random graph that best fits the observed network data. 

In hierarchical random graphs, the probabilities of connecting any two vertices and sub-networks are independent of the presence or absence of other connections. This is similar to the classical Erdos-Renyi random graph. However, in the hierarchical random graph, the probabilities are dependent on the topological structure of the graph.

#### 1) Graph notation

We intuitively model a protein translation network as an undirected graph, where vertices represent proteins and edges represent interactions between pairs of proteins. 

An undirected graph, *G* = (*V, E*), is comprised of two sets, vertices *V* and edges *E*. An edge *e* is defined as a pair of vertices (*i, j*) denoting the direct connection between *i* and* j*. The graphs we use in this paper are undirected, unweighted, and simple – meaning no self-loops or parallel edges.

#### 2) Definition of a hierarchical random graph

Let *n* be the size of vertices set, *n* = |*V*|. Let *D* be the dendrogram with *n* leaves representing vertices of *G*. Let *r* be an internal node of* D* with a probability *P_r_* which denotes the probability that an edge exists between two vertices sharing *r* as their lowest common ancestor in* D*. A hierarchical random graph is thus defined by (*D*, {*P_r_*}).

#### 3) Inferring the hierarchical structure

As stated earlier, one assumption is that the likelihood of all hierarchical random graphs is *a priori* equal. By Bayes’ theorem, the probability that a model (*D*, {*P_r_*}) explains the observed data is proportional to the posterior probability or likelihood *L*. 

Let *E_r_* be the number of edges in *G* with* r* as their lowest common ancestor, *L_r_* and *R_r_* be the numbers of leaves in the left and right subtrees rooted at *r* in *D*. We have

For each internal node *r* in *D*, the probability { } that maximizes *L* is . Thus, the likelihood of *D* at this maximum is

Conveniently, instead of using the above equation directly, we use its logarithm form:

#### 4) Markov chain Monte Carlo method

Since it is an NP hard problem to maximize *L*(*D*, {*P_r_*}), the estimation is done by using a Markov chain Monte Carlo method by sampling *D* with probability proportional to their likelihood. 

With networks of relative small sizes, the Markov chain converges fairly quickly. Therefore, it is suitable for our constructed PTRNs..

## Results 

### Fitting the hierarchical random graph to data

**Figure 1:**
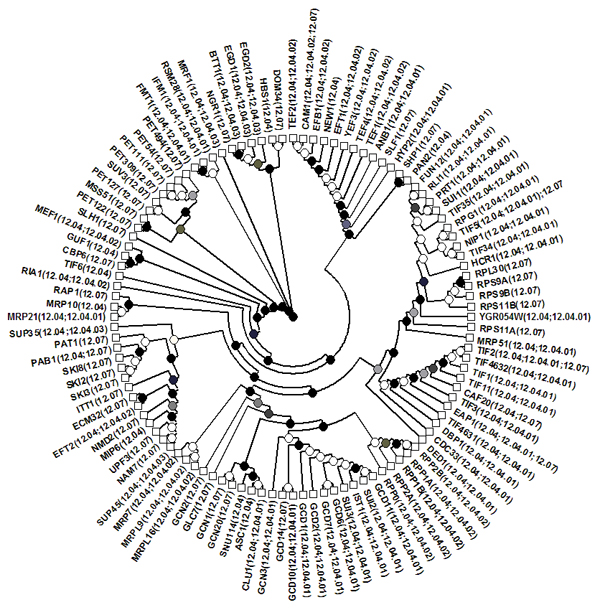
**An example of maximum likelihood dendrogram with *logL*= -539** The leaves are labelled with protein names with corresponding MIPS function categories in parentheses. The probabilities are shown as gray-scale values.

We construct our protein translation network using protein-protein interactions among extracted proteins and then fit the hierarchical random graph model to the constructed network. Fig. 1 shows an example of maximum likelihood dendrogram with *logL* = -539. The dendrogram clearly divides the majority of proteins into groups coherent to their MIPS function categories.

### Consensus dendrogram

**Figure 2:**
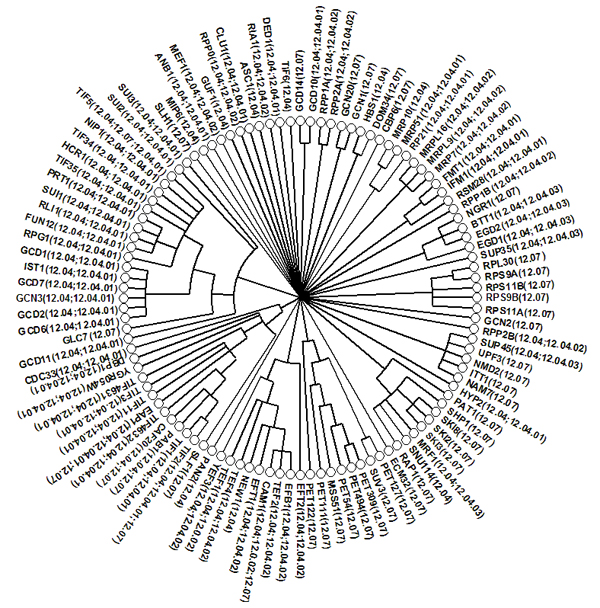
**The consensus dendrogram** The leaves are labelled with protein names with corresponding MIPS function categories in parentheses.

Fig. 2 shows an example of a consensus dendrogram constructed from the sampled hierarchical random graphs. A consensus dendrogram is a summary of a set of dendrograms that fit the observed data. We may expect it to capture the topological features consistent across the majority of the dendrograms and can better characterize the structure of the network than any individual dendrogram.

### Prediction of missing links

The most interesting and possibly the most useful application of hierarchical random graphs is the prediction of missing interactions in networks in which the available information is incomplete as in the case of protein-protein interaction data, especially in our case of studying protein translation regulatory networks. Table 2 is the compiled result of top 15 possible missing links with the highest probabilities from 10 runs of the predicting algorithms. 

**Table 2:** Prediction of missing links

Protein 1	Protein 2	Probability	Reference
SUP35	PAT1	0.8895	[18]
RLI1	PRT1	0.7343	
GCD11	IST1	0.7163	
GCD11	SUI1	0.7161	
GCD11	RLI1	0.7159	
GCD11	HCR1	0.7157	[18]
SUI3	HCR1	0.6977	
SUI3	TIF35	0.6976	
SUI3	FUN12	0.6976	
SUI3	RLI1	0.6976	[18]
SUI3	TIF34	0.6973	
SUI2	FUN12	0.6683	
SUI2	HCR1	0.6682	[18]
SUI2	IST1	0.6681	
SUI2	SUI1	0.6681	

On top of the list is the interaction between SUP35 and PAT1. SUP35 is translation termination factor eRF3, involved in the termination of protein translation. PAT1 is topoisomerase II-associated deadenylation-dependent mRNA-decapping factor. It is required for faithful chromosome transmission, maintenance of rDNA locus stability, and protection of mRNA 3'-UTRs from trimming. There is no interaction between these two proteins in our downloaded datasets. However, this interaction has been reported rather recently [18].

An intriguing finding of the prediction results is that a few proteins have multiple highly probable missing links, such as GCD11, SUI3, SUI2, RLI1, IST1, and HCR1. GCD11 is the gamma subunit of the translation initiation factor eIF2, involving in the identification of the start codon. Its interaction with HCR1 has been reported recently [18]. RLI1 is an essential iron-sulfur protein required for ribosome biogenesis and translation initiation. Its interaction with SUI3 is also reported [18]. SUI3 is the beta subunit of the translation initiation factor eIF2, involved in the identification of the start codon and possibly in mRNA binding as well. HCR1 is a dual function protein involved in translation initiation as a substoichiometric component (eIF3j) of translation initiation factor 3 (eIF3) and is required for processing of 20S pre-rRNA. The interaction between SUI3 and HCR1 has also been reported [18].

## Discussion 

In this paper, we present the exploratory analysis of a protein translation regulatory network using hierarchical random graphs. 

We constructed a protein translation network by extracting proteins categorized in MIPS function database [17] and protein-protein interaction data curated in BioGRID [16]. One important feature of such reconstructed networks is its incompleteness. Our current knowledge about the links may only be a fraction of all interactions among these proteins that may exist in reality. It thus is an enormous challenge to study such partial networks. As shown in Figure 1, by using the hierarchical random graphs, the reconstructed dendrogram divided the majority of proteins into groups corresponding to their MIPS function categories. Our results clearly demonstrated 1) the existence of the hierarchical structure in the constructed protein translation network; and 2) the usefulness of the hierarchical random graph model in exploring the network structure. 

Our results also show the ability of predicting missing links in networks by using the hierarchical random graph. At least four of the top 15 predicted missing links has been reported recently [18]. It is very beneficial for experimental biologists to use such drastically narrowed list to formulate and validate hypotheses. One of our future work will be to collaborate with biologists to validate the predicted missing links and eventually help build up a much more complete translation regulatory network. 

A limitation of current approach using Markov chain Monte Carlo is its high computational cost. Improving the computation efficiency in the future will allow us to apply this approach to larger networks. 

## Conclusions 

In this paper, we apply a hierarchical random graph model in analyzing yeast protein translation regulatory networks. We reconstruct protein translation regulatory networks from a protein-protein interaction dataset. Using the hierarchical random graphs, we show that the reconstructed network exhibits well organized hierarchical structure. Furthermore, we apply this technique to predict missing links in the network. Therefore, the hierarchical random graph mode can be a potentially useful technique for inferring network hierarchical structure and predicting missing links in partly known networks. The results have potential implications for better understanding mechanisms of translational control from a system’s perspective. 

## Competing interests

The authors declare that they have no competing interests.

## Authors' contributions

DDW designed the study, performed the computational experiments, and drafted the manuscript. XH supervised the study. E.K. Park, Xiaofeng Wang, Jiali Feng, and Xindong Wu contributed in the study and experiment. All authors contributed to the manuscript and approved the final version.

## References

[B1] Merrick WC Cap-dependent and cap-independent translation in eukaryotic systems. Gene 2004 332 1 11 15145049 10.1016/j.gene.2004.02.051

[B2] Pain VM Initiation of protein synthesis in eukaryotic cells. Eur. J. Biochem 1996 236 747 767 8665893 10.1111/j.1432-1033.1996.00747.x

[B3] Lockhart DJ Winzeler EA Genomics, gene expression and DNA arrays. Nature 2000 405 827 836 10866209 10.1038/35015701

[B4] de Jong H Modeling and simulation of genetic regulatory systems: a literature review. J Comput Biol 2002 9 67 103 11911796 10.1089/10665270252833208

[B5] Holland EC Regulation of translation and cancer. Cell Cycle 2004 3 452 455 14976427

[B6] Mehra A Lee KH Hatzimanikatis V Insights into the relation between mRNA and protein expression patterns: I. Theoretical consideration. Biotechnol Bioeng 2003 84 822 833 14708123 10.1002/bit.10860

[B7] Mehra A Hatzimanikatis V An algorithmic framework for genome-wide modeling and analysis of translation networks. Biophys J 2006 90 1136 1146 16299083 10.1529/biophysj.105.062521 PMC1367265

[B8] Wu DD Hu X Global analysis of protein translation networks in yeast. Posters of the Computational Systems Bioinformatics Conference 2006 http://lifesciencessociety.org/CSB2006/

[B9] Ravasz E Somera AL Mongru DA Oltvai ZN Barabasi A-L Hierarchical organization of modularity in metabolic networks. Science 2002 297 1551 1555 12202830 10.1126/science.1073374

[B10] Guimerà R Sales-Pardo M Amaral LAN Modularity from fluctuations in random graphs and complex networks. Phys Rev E 2005 70 025101 10.1103/PhysRevE.70.025101 PMC2441765 15447530

[B11] Soffer SN Vazquez A Clustering coefficient without degree correlations biases. Phys Rev E 2005 71 057101 10.1103/PhysRevE.71.057101 16089694

[B12] Sales-Pardo M Guimerà R Moreira AA Amaral LAN Extracting the hierarchical organization of complex systems. Proc Natl Acad Sci USA 2007 104 15224 15229 17881571 10.1073/pnas.0703740104 PMC2000510

[B13] Clauset A Moore C Newman MEJ E.M. Airoldi et al Structural inference of hierarchies in networks. Lecture Notes in Computer Science 2007 4503 Springer-Verlag 1 13

[B14] Clauset A Moore C Newman MEJ Hierarchical structure and the prediction of missing links in networks. Nature 2008 453 98 101 18451861 10.1038/nature06830

[B15] Breitkreutz B-J Stark C Tyers M The GRID: The General Repository for Interaction Datasets. Genome Biol 2003 4 R23 12620108 10.1186/gb-2003-4-3-r23 PMC153463

[B16] Bader GD Betel D Hogue CW BIND: the Biomolecular Interaction Network Database. Nucleic Acids Res 2003 31 248 250 12519993 10.1093/nar/gkg056 PMC165503

[B17] Mewes HW Frishman D Guldener U Mannhaupt G Mayer K Mokrejs M Morgenstern B Munsterkotter M Rudd S Weil B MIPS: A database for genomes and protein sequences. Nucleic Acids Res 2002 30 31 34 11752246 10.1093/nar/30.1.31 PMC99165

[B18] Wilmes GM Bergkessel M Bandyopadhyay S Shales M Braberg H Cagney G Collins SR Whitworth GB Kress TL Weissman JS Ideker T Guthrie C Krogan NJ A genetic interaction map of RNA-processing factors reveals links between Sem1/Dss1-containing complexes and mRNA export and splicing. Mol Cell 2008 32 735 746 19061648 10.1016/j.molcel.2008.11.012 PMC2644724

